# P-713. Two Seasons of Admissions Screening for Influenza and RSV Among a Highly Immunocompromised Patient Population

**DOI:** 10.1093/ofid/ofae631.909

**Published:** 2025-01-29

**Authors:** Robin T Odom, LaToya A Forrester, Angela Michelin, Joseph Scaletta, Alison Han, David K Henderson

**Affiliations:** National Institutes of Health, Bethesda, Maryland; National Institutes of Health, Bethesda, Maryland; NIH Clinical Center, Bethesda, Maryland; NIH Clinical Center, Bethesda, Maryland; National Institutes of Health, Bethesda, Maryland; NIH, Bethesda, MD

## Abstract

**Background:**

To facilitate early detection and prompt isolation of infected patients during the 2022-23 and 2023-24 fall/winter seasons, the NIH Clinical Center, a clinical research hospital serving a significantly immunocompromised patient population, augmented symptom screening with Influenza and RSV testing of all patients on admission.

**Methods:**

Nasopharyngeal (NP) swabs were collected on admission from November 2022-April 2023 and September 2023-April 2024. All samples were tested for Influenza and RSV by PCR on the Panther Fusion® SARS-CoV-2/FluA/B/RSV Assay (Hologic, Inc.) or the Xpert® Xpress CoV-2/Flu/RSV test. Patients who disclosed symptoms on admission were tested using the BioFire® Respiratory Panel. Medical chart reviews and discussions with patient care providers elucidated whether cases identified on admission were asymptomatic.

**Results:**

Of 3197 NP swabs collected from patients admitted to our hospital, 31 swabs were from patients who were symptomatic on admission. Of the remaining 3166 NP swabs (from 2352 unique patients, as some patients had multiple admissions), 17 patients were found to be infected: 7 (0.22%) Influenza A, 1 (0.03%) Influenza B, and 9 (0.28%) RSV. Of these patients that were asymptomatic on admission, 8 (47%) subsequently disclosed symptoms (1 with recent confirmed diagnosis of Rhinovirus/Enterovirus infection), 2 (12%) disclosed a recent history of an unknown respiratory infection, and 7 (41%) were asymptomatic. Seven Influenza A and 8 RSV infections were detected between the months of November and January, yielding positivity rates of 0.57% and 0.66%, respectively, during these months when community transmission was highest.

**Conclusion:**

Admission surveillance for Influenza and RSV during months of increased community transmission allowed us to isolate seventeen patients on admission who would likely not have been appropriately isolated based on symptom screening alone. Admission surveillance testing, in conjunction with symptomatic surveillance, is important to identify infected patients promptly during increased respiratory virus activity in the community, and especially for institutions serving immunocompromised patients.

**Disclosures:**

**All Authors**: No reported disclosures

